# Dynamic Single-Fiber Pull-Out of Polypropylene Fibers Produced with Different Mechanical and Surface Properties for Concrete Reinforcement

**DOI:** 10.3390/ma14040722

**Published:** 2021-02-04

**Authors:** Enrico Wölfel, Harald Brünig, Iurie Curosu, Viktor Mechtcherine, Christina Scheffler

**Affiliations:** 1 Leibniz-Institut für Polymerforschung Dresden e. V. (IPF), Hohe Straße 6, 01069 Dresden, Germany; woelfel@ipfdd.de (E.W.); bruenig@ipfdd.de (H.B.); 2 TU Dresden, Institute of Construction Materials, 01062 Dresden, Germany; iurie.curosu@tu-dresden.de

**Keywords:** polypropylene fiber, drawing ratio, mechanical properties, surface roughness, single-fiber pull-out test, high strength cementitious matrix, quasi-static and dynamic loading

## Abstract

In strain-hardening cement-based composites (SHCC), polypropylene (PP) fibers are often used to provide ductility through micro crack-bridging, in particular when subjected to high loading rates. For the purposeful material design of SHCC, fundamental research is required to understand the failure mechanisms depending on the mechanical properties of the fibers and the fiber–matrix interaction. Hence, PP fibers with diameters between 10 and 30 µm, differing tensile strength levels and Young’s moduli, but also circular and trilobal cross-sections were produced using melt-spinning equipment. The structural changes induced by the drawing parameters during the spinning process and surface modification by sizing were assessed in single-fiber tensile experiments and differential scanning calorimetry (DSC) of the fiber material. Scanning electron microscopy (SEM), atomic force microscopy (AFM) and contact angle measurements were applied to determine the topographical and wetting properties of the fiber surface. The fiber–matrix interaction under quasi-static and dynamic loading was studied in single-fiber pull-out experiments (SFPO). The main findings of microscale characterization showed that increased fiber tensile strength in combination with enhanced mechanical interlocking caused by high surface roughness led to improved energy absorption under dynamic loading. Further enhancement could be observed in the change from a circular to a trilobal fiber cross-section.

## 1. Introduction

Polypropylene (PP) fibers are the most widely used polymer fibers for application in fiber reinforced concrete [1]. They are extensively used in low volume amounts for early age crack prevention [2,3] and to enhance the fracture behavior of concrete in its hardened state [4,5]. PP fibers are also applied in concrete to mitigate spalling at high temperatures [6]. Due to their low melting temperature of circa 170 °C [7], PP fibers melt when exposed to higher temperatures and leave channels through which water vapor pressure is relieved, and in turn, spalling effects are reduced [8,9]. Furthermore, PP fibers are used in concretes and mortars to improve their mechanical resistance to impact loading by increasing their energy-to-fracture and impact resistance [10,11,12].

In general, high-performance polymer microfibers such as polyvinyl alcohol (PVA), ultra-high molecular weight polyethylene (UHMWPE), poly(p-phenylene-2,6-benzobisoxazole) (PBO), and aramid fibers are known to be very effective in strain-hardening cement-based composites (SHCC), also called engineered cementitious composites (ECC). They ensure appropriate crack-bridging for high pre-peak ductility through the formation of multiple cracks under increasing deformation [13,14,15]. In comparison, PP fibers show some weak points, notably their low modulus of elasticity and tensile strength as well as their hydrophobic surface, which strongly limits the bond with the cement matrix. On the other hand, the chemical inertness of the PP fibers leads to excellent durability in corrosive environments, so that they are neither affected by the high alkalinity in the cementitious composites nor by acids and salts [16]. Despite PP fibers’ showing some disadvantages compared to high-modulus polymer fibers with hydrophilic surfaces (as PVA or PBO fibers), their worldwide availability and low cost make them attractive for application in cement-based composites. PP fibers are also manufactured by a more advantageous melt-spinning process, which as well is much less cost-intensive and faster when compared to the slower and more complex wet or gel-spinning processes required for UHMWPE- or PVA fibers [17]. According to Yu et al. [15], in a typical SHCC containing 2 vol. % PVA-fibers (e.g., ECC M45 [18]), 80% of the material costs are attributed to the fibers. Considering the same material basis, the costs of PE-fibers are on top of that eight times higher in comparison with PVA-fibers. Thus, the development of low-cost fibers with adequate crack-bridging is essential to the large-scale applicability and sustainability of SHCC.

Based on this idea, de Lhoneux et al. [19] developed high-tenacity PP fibers with optimized surface properties for improved bonding to concrete matrices. At the same time, recycled fibers receive increasingly more attention in the current research. In the case of PP material, the industry has already established initial recycling routes so that recycled PP is already available for the production of fibers in the low-cost melt-spinning process. To date, no recycling routes exist, however, for UHMWPE or PVA materials since they require more complicated spinning process routes, but also high material purity and quality to achieve their high mechanical performance. In addition to PP fibers, the massive amount of polyethylene terephthalate (PET) used worldwide, leading to landfill and environmental issues, is of interest for the development of recycled PET-fibers for sustainable SHCC made from low-cost plastic wastes [15,20,21]. Low-cost and recycled fibers also show potential in fiber hybridization, meaning the replacement of high-performance and high-cost fibers partly or completely by PP or recycled PET. To be successfully used in future applications, the fundamental interrelationships of the fibers’ structure and surface quality in combination with different concrete matrices, but also under different loading scenarios, is of great importance. SHCC reinforced with polymer fibers yields pronounced strain-rate dependent tensile behavior; however, the failure mechanisms that lead from fiber pull-out to fiber rupture are not fully understood. Therefore, the pull-out tests in this study were conducted at different loading rates to investigate the relationships between fiber morphology and mechanical properties on the micromechanical failure behavior at different pull-out velocities.

Polyolefin fibers used in cementitious matrices are available in various shapes and geometries depending on their manufacturing and function in the concrete composite. They can be distinguished in PP monofilaments, fibrillated PP microfibers and macro-synthetic PP fibers according to their diameter, length, and cross-sectional shape. The latter lead to different tensile strengths (micro: 150–400 MPa; fibrillated: 300–500 MPa; macro: 470–690 MPa) and moduli of elasticity (micro: 2–4 GPa; fibrillated: ≈5 GPa; macro: 5–10 GPa). A detailed description dealing with the manufacturing processes, properties, modifications, and their application was summarized in 2016 by Zych and Krasodomski [22].

**Figure 1:**
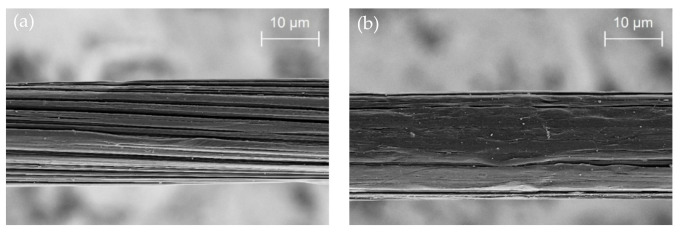
Examples of surface structures of ultra-high molecular weight polyethylene (UHMWPE) fibers, (**a**) high surface roughness by fibrillated surface resulting from high draw ratios during fiber manufacturing, (**b**) part of the surface with less pronounced fibrillation and reduced roughness.

To improve the weak interfacial bonding of PP fibers, two different strategies can be followed: (i) increasing the mechanical interlocking between fiber surface and concrete and (ii) treating the surface to improve the chemical interaction. Described are various types of fiber geometries that enhance fiber anchorage in the matrix, such as crimped fibers, twisted fibers, fibers with enlarged, sinusoidal or hooked ends [22,23], or flat fibers with indented/embossed surface structures [24]. The chemical inertness of PP results in the lack of any functional group that could chemically interact with the concrete matrix. To overcome this problem, functional groups could be either introduced into the polymer material itself that is spun to the fiber or in an outer surface layer, which is in contact with the concrete matrix. In the case of macrofibers, core/sheath-structures have been developed, and these types of fibers are already established on the market; the fiber producers are listed in [22]. Additionally, in many cases, the bicomponent structure of the fiber is accompanied by geometrical structuring for stronger mechanical interlocking [25]. A combination of the described approaches was also used in [19]: a bicomponent PP fiber was developed with pure PP in the core and a PP compound in the sheath-layer with an additional surface treatment that was applied after spinning. However, no information has been given respecting the functional groups that were introduced to the PP compound or the substances applied by the fiber finish. In comparison to the macrofibers with diameters ranging between approx. 800 and 1100 µm, microfibers with diameters from 15 to 50 µm are mainly spun as mono-component fibers [22]. The increase of the chemical bond by the addition of compounds to the PP, but also the spin finish or surface treatment by coating or plasma [21,26] are feasible for enhancing the interfacial interaction. The application of silane on the surface of steel fibers was proven to enhance the mechanical and chemical interaction between the fiber and the matrix material [27,28]. This approach also seems conceivable for PP fibers. Although the structuring of the fiber surface geometry is a widely used method to increase the mechanical anchorage of macrofibers, this approach is rarely followed for microfibers due to their small diameters and high spinning velocity. However, polyolefin microfibers also reveal a surface roughness on the micro- or nanoscale resulting from the drawing process; see Figure 1. However, its contribution to the processes during debonding, pull-out, and fiber failure is sparingly described in the current literature.

Single-fiber pull-out (SFPO) tests are an established method for the characterization of the fiber–matrix bond. Since the 1980s, numerous experimental test setups and modeling approaches have been proposed to describe the processes responsible for debonding, pull-out, and fiber rupture as related to different fiber materials and their interfacial bond behavior [29,30,31]. Particularly, SFPO on steel fibers with different geometries [32], considering fiber orientation [33], surface modifications, e.g., silane [28], type of concrete matrix [32,34] and loading rates [35,36] were conducted, and many data have been published to date. Except for the work done by de Lhoneux et al. in [19], where bicomponent PP fibers were spun by a pilot spinning line, no literature that includes the systematic variation of PP fiber properties by spinning and drawing, characterization of the resulting surface structure, and pull-out behavior in concrete matrices is as yet available. This work aims to fill this gap by the production of PP fibers with melt-spinning equipment and applying different draw ratios to the fibers, hence leading to different strain capacities and moduli of elasticity. The PP fibers’ mechanical properties and surface characteristics are investigated with regard to contact angle, surface roughness, and the resulting interaction with concrete matrix studied in single-fiber pull-out tests under quasi-static and dynamic loading.

## 2. Materials and Methods

### 2.1. Fiber Production and Surface Treatment

The polymer fibers investigated were manufactured at IPF with a laboratory-scale melt-spinning device, which allows the variation of the spinning parameters within wide ranges; the setup used is described in [37]. For all spinning trials, a melt temperature of the polypropylene, granule-type HG475FB, homopolymer supplied by Borealis, was maintained constant at 235 °C. For the production of fibers with diameters of 10/20/30 μm, a spinneret with 48/24/12 holes was used; see Table 1. The capillary diameter of the holes was 0.25 mm, and the length/diameter ratio L/D was 2. The mass throughput of the spinning pump varied between 7.2 and 21.6 g/min. For investigations of the influence of a more complex cross-sectional shape, trilobal fibers with an equivalent diameter of 20 µm were produced. The equivalent diameter corresponds to the diameter of a circular fiber with the same cross-sectional area. During the online stretching process, the draw ratio was adjusted in the range from 1.00 to 2.25, and the winding speed between 1500 and 3400 m/min.

Since it was aimed to study the fiber pull-out behavior depending on the fiber’s mechanical properties and shape, the application of a spin finish was avoided. Instead, the fiber drawing was performed using pure water. Additionally, to gather information on the effect of a subsequent sizing on the interaction between fiber and the cementitious matrix, sizing was applied via a special dip-coating procedure. Single fibers were fixed on a frame and dipped at a constant velocity into the sizing, designated as PP sizing, which consists of a PP film former, AQUACER 598 supplied by BYK-Chemie GmbH, Wesel, Germany, and 3-aminopropyl-triethoxysilane, Dynasylan^®^ AMEO supplied by Evonik Industries, Marl, Germany, as an adhesion promoter. Subsequently, the modified fibers were dried at room temperature, followed by further treatment in a convection oven at 60 °C for 30 min each.

### 2.2. Concrete Matrix

The fibers were combined with a high-strength cementitious matrix based on Portland cement as the main binder. The composition of the mixture is given in Table 2. Curosu et al. [38] described this high-strength SHCC-matrix in detail in previous studies. The mixture was selected because of its low maximum aggregate size of approximately 200 µm and its high packing density, which is advantageous for the anchoring of the single fibers.

### 2.3. Fiber Surface Characterization

Contact angle measurements were carried out to investigate the wetting behavior of the fiber surface. Therefore, a tensiometer, DCAT 21 (DataPhysics Instruments GmbH, Filderstadt, Germany), was used to perform the measurement by dipping single fibers into pure water. The determination of the advancing angle was conducted with the help of the Wilhelmy method [39].

The fiber surface was examined using a scanning electron microscope ULTRA PLUS (Carl Zeiss Microscopy GmbH, Jena, Germany) equipped with an secondary electron (SE2) detector. The fiber surface was studied after production in the initial state but also after being pulled out of the cement-based matrix to assess the surface damage.

The surface roughness of the fibers in their initial state was determined by atomic force microscopy (AFM) using a Dimension Icon (Bruker Corporation, USA) equipped with a Tap300-G BudgetSensors^®^ (Innovative Solutions Bulgaria Ltd., Bulgaria) cantilever made of monolithic silicon with a rotated pyramid-shaped tip and a tip radius of less than 10 nm. Topography images with 3 µm × 3 µm and 512 px × 512 px were recorded in the tapping mode. The determination of the roughness parameters. *R_a_* (arithmetic average roughness) and *R_max_* (maximum roughness)—the difference between the lowest groove and the highest top—was carried out based on 5 images for each fiber type.

### 2.4. Mechanical Testing of the Fibers

Single-fiber tensile tests were conducted with a FAVIMAT+ (Textechno Herbert Stein GmbH and Co.KG, Mönchengladbach, Germany) equipped with a 610 cN load cell. For each fiber type, 50 single filaments were tested at a clamping length of 10 mm and a velocity of 5 mm/min. The linear density of every single fiber needed for the evaluation was determined before the tension test using the vibroscopic method according to ASTM D 1577 [40]. Subsequently, the diameter of each fiber was calculated from the linear density using a density of 0.9 g/cm^3^ for PP. The tensile tests were conducted in a standard room climate with an air temperature of 23 °C and relative humidity of 50%.

### 2.5. Differential Scanning Calorimetry

Differential scanning calorimetry (DSC) was used to investigate the influence of the different spinning parameters on the crystallization properties of the PP fibers produced. The samples prepared were measured in a nitrogen atmosphere with a DSC Q2000 (TA Instruments, USA). The samples were heated at a rate of 10 K/min from −50 to 200 °C (1st run), cooled down at the same rate and heated again (2nd run), during which the heat flow was recorded. The determination of crystallinity was done using a melting enthalpy for the polypropylene of ∆H_lit_, 100% = 207 J/g, according to [41].

### 2.6. Single-Fiber Pull-Out Tests (SFPO)

**Figure 2:**
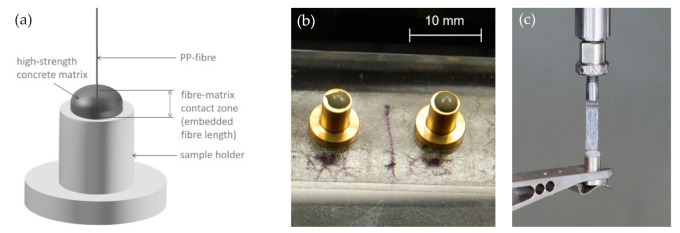
(**a**) Schematic single-fiber model composite, (**b**) real specimens with high strength matrix and (**c**) setup of the dynamic single-fiber pull-out experiments (SFPO).

For the preparation of single-fiber model composites as well as for the fiber pull-out, an in-house constructed device was used (Figure 2) [42,43,44]. The preparation of the cementitious matrices was performed by stirring the components with a speed mixer. The mixture was then filled into a sample holder of cylindrical shape and an inner diameter of about 2.6 mm (Figure 2b). The matrix droplet formed, therefore, also had a cylindrical body with a hemispherical shape on top. After transferring the matrix-filled sample holder to the embedding device, one single fiber was embedded over a length of 1 mm by a computer-controlled procedure. The embedding process was assisted by a two-camera-system, which enables the placement of the fiber on the top of the matrix droplet and the observation of the fiber when penetrating the matrix. The fiber is embedded at a constant velocity. The real embedded length is measured after the pull-out test on an optical microscope. Each specimen produced was stored in a humid atmosphere for 28 days over a desiccator bottom filled with water until testing.

The single-fiber model composite was placed in the pull-out device, and the fiber end was fixed at the mandrel with a cyanoacrylate adhesive in such a way that the free length was minimized; see Figure 2c. Subsequently, the pull-out test was performed under quasi-static loading conditions (QSFPO) at a pull-out velocity of 0.001 mm/s and under dynamic loading (DSFPO) at 10 mm/s, respectively. For the dynamic pull-out test, an updated setup compared to the setup described in [37] was used, which also consists of a piezo actuator, but enables higher maximal displacements up to 450 μm. During the test, the full force–displacement curve was recorded in the case of QSFPO. During DSFPO, the curves were analyzed up to a maximum displacement of 300 µm to ensure a constant strain rate during testing. The limitation of 300 µm is related directly to the linear operating range of the piezo actuator. At least 15 specimens for each fiber type were tested in Q- and DSFPO to ensure meaningful statistical evaluation.

## 3. Results and Discussion

### 3.1. Mechanical Properties of the Fibers

**Figure 3:**
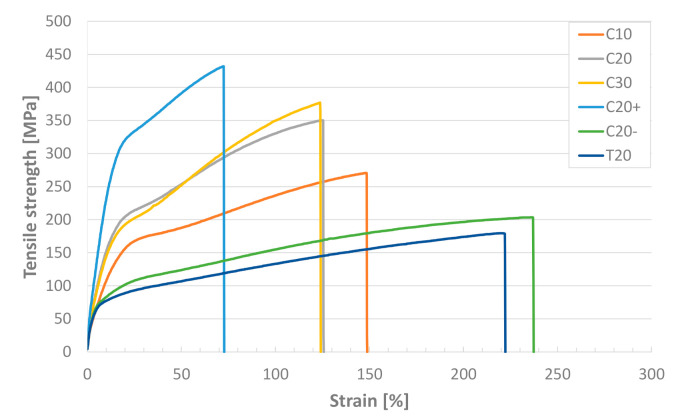
Tensile stress–strain curves of all polypropylene fibers under investigation (C—circular, T—trilobal; 10/20/30 diameter in µm, +/− in−/decreased stretching during production).

The availability of the polymer melt-spinning line enabled the desired adjustment of mechanical properties, diameter, cross-sectional shape, and surface finish of the fibers. The main goal of this study is to clarify the influence of these parameters on the energy absorption and on the damage mechanisms during fiber pull-out, which clarification can be scarcely achieved by using commercially available fiber materials. It was one task to increase the surface contact area by increasing the perimeter while keeping the cross-sectional area and the mechanical properties of the fiber almost identical. This could be achieved by adapting the drawing ratio, here also referred to as “degree of stretching”, during spinning. In this way, similar mechanical properties were reached for the fibers C20 and C30. Typical stress–strain curves of the polypropylene fibers produced and investigated are presented in Figure 3, and the related results in Table 3. For fiber C10 a target diameter of 10 µm was envisaged; however, the drawing parameters were at the limit of what was feasible with the material used. This resulted in a slightly higher diameter of about 13 μm, lower Young’s modulus and tensile strength, and higher residual strain as well.

In addition to diameter variation, the surface contact area was increased by spinning trilobal- instead of circular-shaped fibers to improve the anchoring in the cementitious matrix. Compared to a circular fiber with a diameter of 20 µm and a circumference of approx. 63 µm, the circumference of trilobal fibers with 106 µm, determined using a digital microscope, is considerably higher. The spinning conditions that are applied to achieve the trilobal shape do not permit high degrees of stretching so that the trilobal T20 fibers exhibit the lowest mechanical characteristic values of all the spun fibers.

As another point of interest, the effect of increasing tensile strength on the fiber pull-out behavior was part of the investigation. This was achieved by spinning 20 μm fibers with increasing drawing ratios and goes along with reduced strain capacity. The increased stretching during the production causes the alignment of the polymer chains in the axial direction, which leads to increased anisotropy, induced crystallization, and therefore superior mechanical properties [45]. According to the drawing ratio given in Table 1, C20+ fibers with the highest stretching degree reveal the highest Young’s moduli (4.6 GPa) and tensile strength values (427 MPa), far above the values of C20− fibers with the lowest stretching degree, i.e., Young’s modulus of 2.6 GPa and tensile strength of 195 MPa; see Table 3 and Figure 3. Consequently, the elongation at break is reduced significantly, from approximately 229% for C20− to 76% due to the orientation of the polymer chains along the fiber axis.

In contrast to commercially available PP fibers, no sizing (spin finish) was applied as surface treatment during the spinning process with the aim of excluding sizing influences. This guarantees that the effects observed during single-fiber pull-out tests are related to the geometrical and structural changes and less to physical-chemical effects. Therefore, all fibers were spun using pure water as “lubricant”, which limits the attainable mechanical values for the different shaped fibers because the online drawing was restricted or impossible. An unstable spinning process was especially observed for the trilobal fibers T20 and the very thin fibers C10. The yarn was spread into single filaments due to the absence of yarn cohesion. This effect is avoided commonly by the application of a spin finish (sizing) before the stretching in the spinning process.

### 3.2. Surface Structure and Roughness

**Figure 4:**
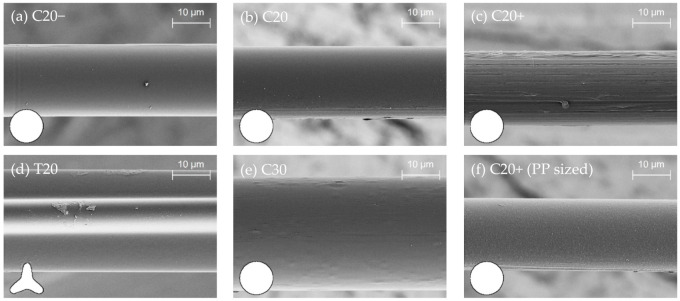
SEM images of spun circular fibers with a diameter of 20 µm showing PP fibers with (**a**) low, (**b**) medium and (**c**) high mechanical properties; (**d**) trilobal fibers, (**e**) circular fibers with the increased diameter of 30 µm and (**f**) PP-sized fiber.

The stresses applied on polymer fibers during melt-spinning with high drawing ratios are known to induce roughening on the surface, e.g., this is described for both low- and high-density polyethylene [46]. The smooth surface forms fibrillary structures, which arise from the deformation of nodular structures caused by bulk spherulites on the surface. Similar behavior was observed for the PP fibers with increasing degrees of stretching using SEM; see Figure 4. The surface of the fibers produced with low and medium stretching stresses C20− and C20 was predominantly smooth. In comparison, C20+ fibers exhibited surface grooves and a fibrillar texture in the fiber direction, leading to higher roughness.

With increasing drawing ratios, a rising number of grooves and deformations on the fiber surface were observed. However, the stretching process did not result in the homogenous formation of fibrillary texture along the entire fiber length and for all fibers. Smooth fibers were also observed for C20+ (not shown). C30 fibers were spun with a drawing ratio similar to C20 and showed mainly smooth fiber surfaces, occasionally with small bumps and very fine grooves. Accordingly, the trilobal fibers T20 also showed a smooth surface because no online drawing could be applied; see Figure 4d. In part, some small deformations on the outstanding sections could be observed because these parts of the complex cross-sectional shape are not particularly resistant to mechanical stresses in comparison to the compact bulk. In the case of the dip-coated C20+ fibers, it can be seen that the entire surface was homogeneously covered with the PP sizing; see Figure 4f. The fine grooves were filled up with the sizing material while the “macro” grooves are still visible.

**Figure 5:**
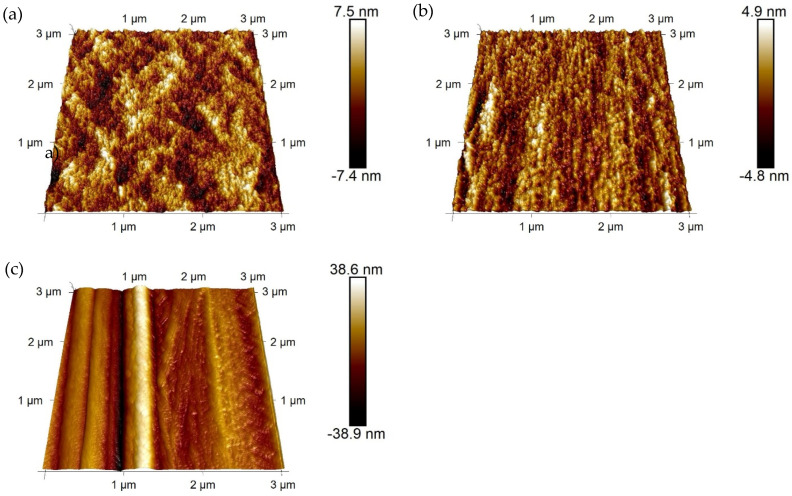
Height images of (**a**) C20−, (**b**) C20 and (**c**) C20+ PP fibers determined by atomic force microscopy (AFM) (tapping mode).

In addition to SEM, a quantitative analysis of the micro-roughness was performed by AFM. The recorded height images of the fibers with low (C20−), medium (C20) and high (C20+) drawing ratios are displayed in Figure 5; the R_a_ and R_max_ values determined are given in Table 4. The average roughness R_a_ and maximum roughness R_max_ were found to be very similar for C20− and C20 fibers. These values are clearly lower compared to C20+ fibers, which reveal values nearly twice as high for R_a_ and R_max_ and confirm the increased roughness due to higher stretching during the production. The characteristic fibrils in the direction of the C20+ fibers are clearly visible in the AFM images. The fibers C20− and C20 exhibit notably high standard deviations because the surface was not homogenously stressed during spinning, as already observed by SEM.

### 3.3. Contact Angle

The wettability of the fibers in water-based, cementitious matrices plays a crucial role during the composite formation process. Since the predominantly inorganic matrix has a polar character, pure water was selected as the polar liquid to be used in the contact angle measurements. The advancing contact angles of the PP fibers formed against water are given in Table 5. The advancing angles are above 90° and correspond to literature references, e.g., [47]. The contact angles highlight the nonpolar character of the PP fibers and indicate poor wetting in contact with the cementitious matrices. Nevertheless, the values vary in a wide range from approx. 90° for C20+ to approx. 105° for C20, which is related to non-uniformity of the fiber surface roughness as discussed in Section 3.2 [48]. In addition, the PP fibers tend to bow during sample preparation for contact angle measurements, which could be observed predominantly for some of the thin C10 and highly stretched C20+ fibers. The application of the polypropylene sizing, sample C20+ (PP-sized), via dip-coating leads to a minor decrease of the contact angle to approximately 88°. The slightly improved wetting can be attributed to the silane and surfactants contained in the PP film former.

### 3.4. Crystallinity

The chain alignment in the PP material induced by the differing drawing ratios and stretching goes along with the crystallization effects investigated using differential scanning calorimetry (DSC). Crystallization in melt-spinning processes is described in detail in the literature [45,49]. It is known that the high cooling rate in melt-spinning enables crystallization, which occurs only during a short time period. The crystallization is very sensitive to orientation and stress, so that spinning with an increasing take-up velocity results in higher density due to the attendant increased crystallization.

**Figure 6:**
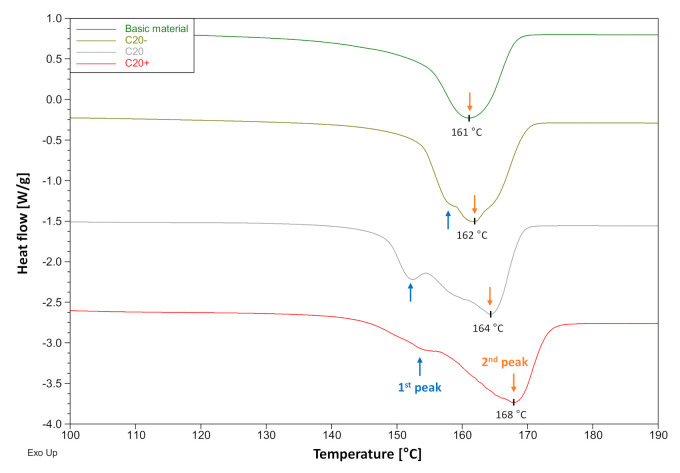
Influence of the drawing parameters on crystallization behavior during melt-spinning; comparison of the melt peaks determined by differential scanning calorimetry (DSC) for basic PP material (2nd heating), and PP fibers C20−, C20 and C20+ with increasing draw ratio (1st heating).

As a reference, the basic PP material in the form of granules was used to analyze the melting enthalpy values and peak temperatures during second heating; see Figure 6 and Table 6. The thermograms of the PP fibers with different stretching degrees C20−, C20, and C20+ were assessed during the first heating. With increased stretching of the fibers, the peak melting temperature of the crystals rose from 162 °C for C20− to 168 °C for C20+. The melting temperature of the basic material without chain alignment was found to be lowest at 161 °C since no stress-induced crystallization occurs. The cooling rate of PP material in melt-spinning is extremely high when compared to PP bulk materials; literature values of up to >1000 K/s have been specified [50] but were not expected to be as high in this spinning program. The high cooling rate and the short time window, which allow for the chain alignment, led to various sizes and types of the formed crystallites, which resulted in multiple different peaks during melting, especially in the case of C20 and C20+. Additionally, the online drawing of the fibers took place when the material was already cooled down, and the chain mobility was low. The drawing forces a further orientation of the chains that come closer to each other, leading to new, small, imperfect arrangement sections. These melt at lower temperatures during heating; see the widening of the 1st peak to lower temperatures in Figure 6. The already aligned sections are further densified by the additional drawing, which is associated with increased melting enthalpies and high peak melting temperatures, i.e., second peak.

### 3.5. Quasi-Static and Dynamic Fiber Pull-Out

The characterization of the fiber structure and surface properties revealed differences in the manufactured PP fibers’ diameter, mechanical properties, and cross-sectional shape. In the next step, the fibers were embedded in concrete matrices in order to produce single-fiber model composites for the SFPO tests. During sample preparation, it became apparent that C10 fibers could not be embedded into the matrix material. Their low structural stiffness caused fiber bending when penetrating the matrix droplet so that the fibers had to be excluded from the SFPO test.

**Figure 7:**
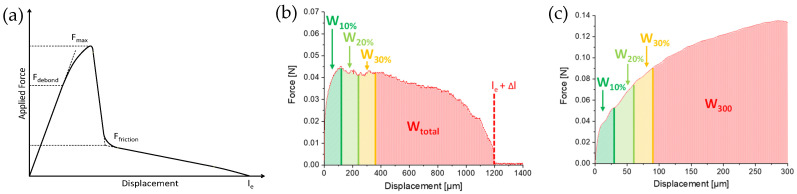
Force–displacement curves in the single-fiber pull-out tests on PP fibers: (**a**) typical curve showing debonding initiation, complete debonding, and frictional pull-out; typical curves were generated in (**b**) quasi-static and (**c**) dynamic tests.

Interfacial debonding in pull-out tests is induced by crack propagation along with the embedded fiber with frictional slip in the debonded regions [51]. The curves show a kink in the ascending part of the force–displacement curve corresponding to debonding initiation (F_debond_, Figure 7a). This force value is the basis for the description of the interfacial adhesion in analytical approaches [51,52]. The applied force then reaches a peak value (F_max_), after which the interfacial adhesion and friction are no longer in balance, causing uncontrolled debonding until the frictional bond is reached. This is indicated by a characteristic force drop. From this point (F_friction_) to full fiber pull-out, the measured force value is determined by fiber–matrix friction.

**Figure 8:**
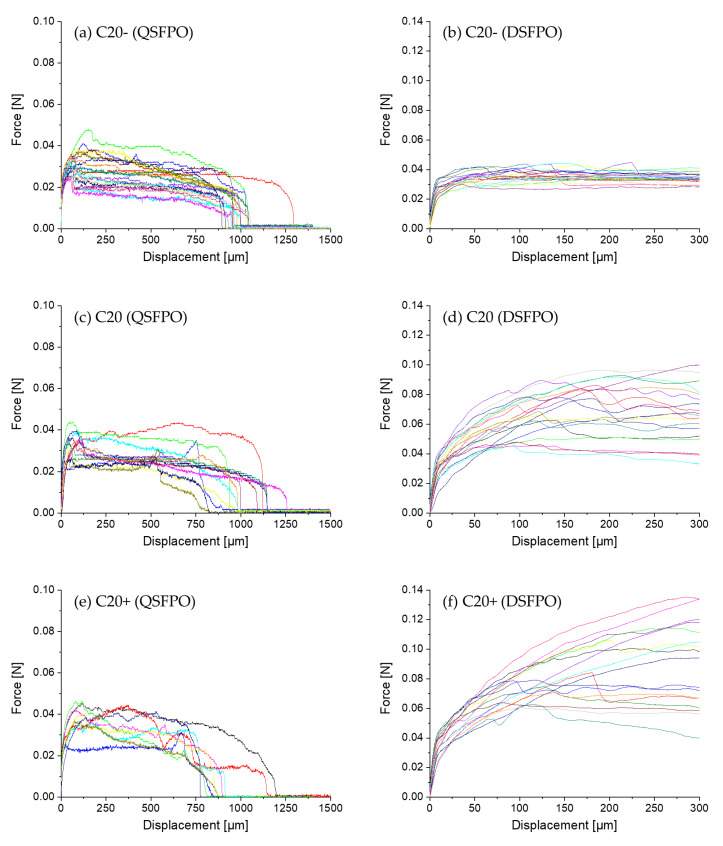
Force–displacement curves recorded during single-fiber pull-out of (**a**,**c**,**e**) circular C20−, C20, and C20+ fibers embedded in high-strength concrete matrix under quasi-static and (**b**,**d**,**f**) dynamic loading. Note the different axes’ limits. Color lines indicate different test samples.

**Figure 9:**
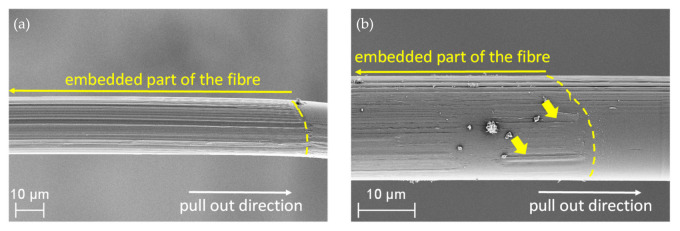
SEM images showing deformations on PP fiber surfaces for (**a**) fiber C20 after quasi-static pull-out and (**b**) fiber C20+ after dynamic pull-out, the arrows highlight grooves caused by surface abrasion.

A comparison of the force–displacement curves of C20−, C20, and C20+ fibers embedded in the high-strength matrix and pulled-out under quasi-static and dynamic loading is presented in Figure 8. It becomes obvious that for this fiber–matrix material combination, the force–displacement curves do not follow the characteristic stages observed during fiber pull-out [29,43,53]. The characteristic points F_debond_ or F_friction_ needed for evaluation cannot be determined because of different overlapping effects. The pull-out force depends on both chemical interactions and friction due to mechanical interlocking. Additionally, in the case of polymer fibers, the fiber elongation and the plastic deformation of the fiber surface contribute to the force values measured. Furthermore, the setup for DSFPO does not allow a displacement of more than 300 µm, so that the real embedded fiber length, commonly used to calculate the interfacial parameters, cannot be derived from the curves. On the other hand, several fibers of C20−, C20, but also C20+ fibers, were remarkably stretched during QSFPO, which becomes clear by a shift of the end of the pull-out curves towards higher displacements. In order to compare the results of both loading rates, the pull-out work W_total_ and W_300_ were determined; see Figure 7b,c using Equations (1) and (2). W_total_ can only be calculated for the QSFPO and corresponds to the work performed until full fiber pull-out. In contrast, the W_300_ value was determined for the DSFPO and QSFPO to be between zero and 300 µm displacement. W_total_ and W_300_ were calculated for each measured force–displacement curve. The average values for each measurement series are given in Table 7. The values of W_total_ and W_300_ are not only related to the chemical and frictional bonds but also to polymer fiber stretching and to abrasion of the polymer material, as indicated by scratches and grooves on the pulled-out fibers in Figure 9.

(1) $$W_{\mathrm{total}}=\int_{0}^{l_{e}}F\left(s\right)ds$$

(2) $$W_{300}=\int_{0}^{300\;µm}F\left(s\right)ds$$

In addition to the calculation of the pull-out work up to 300 µm displacement and full pull-out at embedded length l_e_, each individual force–displacement curve was divided into ten parts of equal size, depending on the displacement. The pull-out work was determined for each part; see Figure 7b,c. This standardization regarding displacement allows the direct comparison of the evolution of pull-out work along with the pull-out degree for different fiber types; see Figure 11a,c. The further standardization of the calculated pull-out work values concerning W_total_ for quasi-static and W_300_ for dynamic loading (W/W_total_ or W/W_300_) enables direct comparison of the individual work proportions over the pull-out process among different fiber types; see Figure 11b,d.

The force–displacement curves of fibers C20−, C20, and C20+ reveal great differences in their shapes and peak forces under dynamic loading; see Figure 8b,d,f. The overall force level increases with the increasing draw ratios of the fibers such that the values of W_300_ for C20+ exceed the values determined for C20− and C20 fibers; see Figure 11c, Table 7. This trend is also found in the QSFPO; see Figure 8a,c,e, Figure 11a, and Table 7. Since the PP material and the chemical composition of the fiber surface (no sizing) was the same for all fiber types, the differences can be mainly attributed to the increase of the mechanical properties, but also to the higher surface roughness of the C20+ fibers; see Figure 4c and Figure 9. Further, it was recognized that the force–displacement curves of C20+ fibers split into two groups; (i) the smooth and hydrophobic surface of the fibers led to a pull-out with a nearly constant force; (ii) in comparison, higher roughness allowed increased mechanical interlocking and yielded continuously rising forces till the end of data collection. This slip-hardening behavior is beneficial for improved energy absorption not only in single-fiber pull-out tests but for multiple cracking in SHCC. It was found for several fibers that the mechanical interlocking led to plastic deformations of the surface, stretching of the fiber and therefore improved energy absorption during SFPO (Figure 9). In the literature, similar damage and stretching effects due to high surface roughness were reported for UHMWPE fibers [14,38], and in combination with an additional good chemical adhesion to the matrix material for PVA fibers [54,55]. Since the AFM analysis, as shown in Figure 5, Table 4 revealed comparable roughness between the C20 and C20− fibers, the fibers mainly differ in their drawing ratio; so, the higher force level for C20 fibers during pull-out can be traced back to their higher Young’s modulus. The higher Young’s modulus resulted in a reduced radial contraction of the loaded fiber and, consequently, in a less pronounced radial deconfinement at the fiber–matrix interface.

The results contribute to the findings discussed by other research groups for various fiber types, e.g., UHMWPE, PVA and PP [38,56], showing that increasing loading rates result in increased pull-out resistance and work capacity. Additionally, we could clearly relate this increase to the mechanical fiber strength and surface structuring arising from chain alignment by fiber drawing.

**Figure 10:**
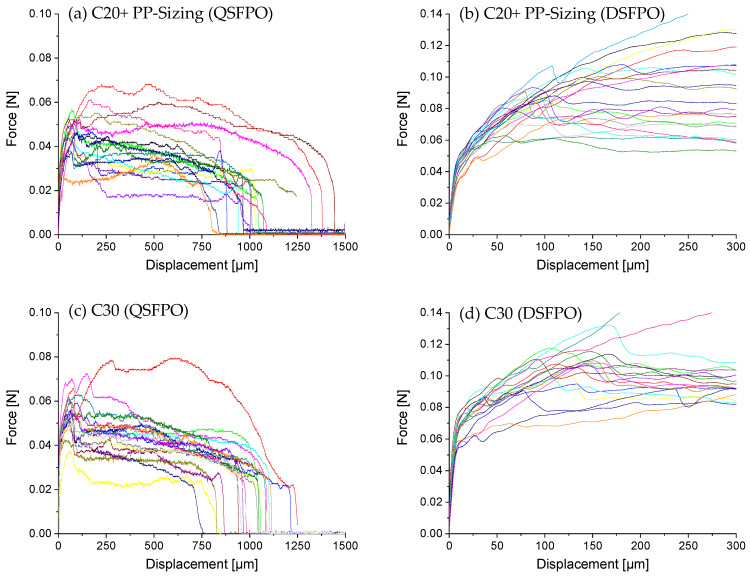

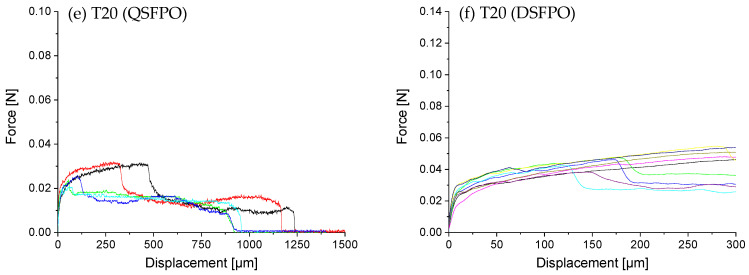
Quasi-static (left) and dynamic (right) SFPO force–displacement curves of (**a**,**b**) C20+ fibers with additionally applied PP sizing dried at 60 °C; (**c**,**d**) C30 fibers and (**e**,**f**) trilobal T20 fibers pulled out of the high-strength matrix. Note the different axes’ limits. Color lines indicate different test samples.

**Figure 11:**
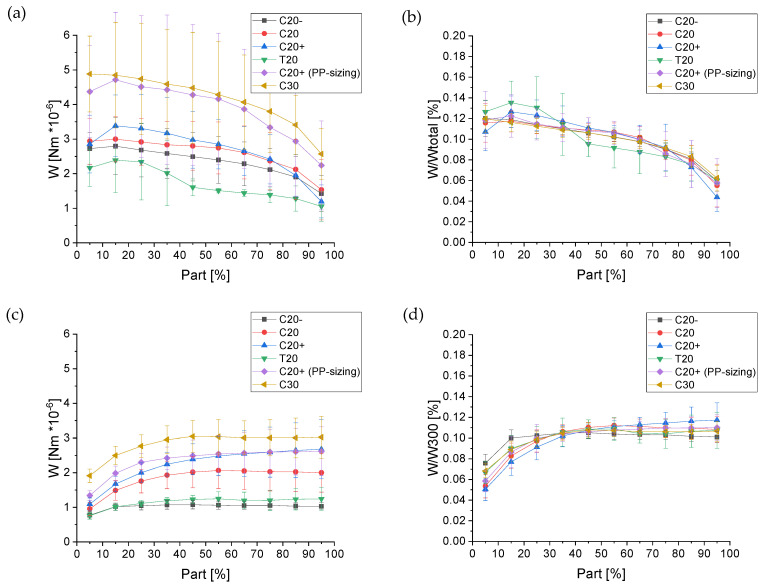
Comparison of (**a**) average progress and (**b**) normalized pull-out work for quasi-static load and (**c**) average progress and (**d**) normalized pull-out work for dynamic SFPO.

C20+ fibers were selected to apply an additional sizing in order to improve wetting behavior to cover the fibrillated surface and to provide at least a minor number of chemical groups to enhance the fiber–matrix interaction. As shown in Section 3.3, the value of the contact angle changed only marginally since PP was used as the film-forming agent. Especially for QSFPO, a strong increase of the pull-out work, as can be seen in Figure 11 and Table 7, in comparison to the unsized fibers, was observed; also, for DSFPO, a beneficial effect was determined; see Figure 10a,b. As displayed in Figure 4, comparing 4c,f, the PP sizing was largely filling the groves on the surface, hence leading to fewer opportunities for mechanical interlocking. Therefore, it can be concluded that sizing plays a major role in enhancing pull-out performance.

In further tests, fibers having a diameter of 30 µm instead of 20 µm, while maintaining the mechanical characteristics at the same level, were used to prepare single-fiber model composites; see Table 3. The force–displacement curves of QSFPO and DSFPO are shown in Figure 10c,d and the pull-out work in Figure 11 and Table 7. The overall force level of C30 fibers was higher in the DSFPO measurements as compared to that of C20 fibers. Specifically, the forces of the first part of the curves have clearly increased. As already found in the previously presented tests, the mechanical fiber properties strongly contribute to the force level achieved during pull-out. Here, in addition to the larger embedded fiber surface area and by extension, the respective fiber–matrix contact area provides for enhanced load transfer, and higher tensile forces are carried by C30 fibers. The same behavior was observed for quasi-static loading, where the force level and pull-out work of C30 exceeds that of the C20 fibers. However, for the application of thicker PP fibers in SHCC, the fiber volume content must be considered. This reveals that the volume of the C30 fiber was more than twice as large as that of the C20 fibers, but the force level and energy absorption capacity is not twice as high. Hence, for the equal fiber volume fraction in the concrete composite, more than twice as many C20 fibers can be used, which results in a larger collective embedment area and denser fiber dispersion, which is essential for proper crack-bridging.

Due to the absence of chemical bonding of the PP fibers to the matrix, many efforts are made to use the potential of mechanical interlocking, e.g., for macrofibers [22,23,24]. Instead, the structuring of microfibers needs to be carried out during the continuous melt-spinning process, which implies that the fibers can only be structured in the fiber direction. In this way, fibers with trilobal cross-sectional shape were also spun in order to study the influence of the more complex cross-sectional shape and increased surface area on the pull-out behavior. The mechanical properties of the trilobal T20 fibers are comparable to those of circular C20− fibers, as seen in Table 3; also, the surface shows no notable structuring in both cases; see Figure 4d. Comparing the force–displacement curves of the C20− fibers during DSFPO, as in Figure 8b, which show nearly constant force up to maximum displacement, the curves of T20 fibers, as in Figure 10f, reveal an increasing pull-out resistance at higher displacements. In addition, the curves are split into two groups: one group is characterized by a constant force increase, whereby the maximum forces exceed the forces achieved for the circular C20− fibers. The second group is indicated by a drop in force at displacements between 120 and 200 μm, with subsequent stabilization at a constant force level. For quasi-static tests of T20 fibers, an overall reduced force level is found, as seen in Figure 10e and Figure 11, but again some curves are characterized by sudden drops in force, occurring in this case between 300 and 500 μm displacement accompanied with a full pull-out length of >1200 μm. This length is clearly greater than the embedded length of 1000 μm used for sample preparation, thus indicating an elongation of the fiber during pull-out. It is therefore assumed that just the upper part of the fiber debonds and is stretched afterward due to the increasing pull-out force; the lower fiber part is still embedded and mechanically anchored. Finally, the fiber diameter decreases and full debonding occurs, as recognized in the force drop. From this point, fiber–matrix friction dominates. In summary, it can be deduced that, in general, an enhanced anchorage in the matrix was achieved for trilobal fibers during QSFPO and DSFPO, but the fibers suffer from their low drawing ratio, which does not provide sufficient mechanical properties. The surface structuring in fiber direction is not effective enough to result in a remarkable increase of pull-out work, as apparent from Table 7, but shows the potential of mechanical anchoring as a factor to affect crack-bridging behavior positively.

Despite the different failure patterns revealed by the force–displacement curves, the evolution of the pull-out work is nearly identical for the C20−, C20, C20+ (PP sizing) and the C30 fibers under quasi-static loading; see Figure 11b. No slip-hardening was observed. On the contrary, under dynamic load, an increase of the pull-out work occurs, culminating in a plateau. Only the C20+ fibers revealed an increase along the entire displacement range from part 10 to 100%; see Figure 11d.

## 4. Conclusions

Young’s modulus, tensile strength, and strain capacity of PP fibers control their pull-out behavior and the related energy absorption under load. Surface roughness leads to mechanical interlocking and, therefore, to plastic deformation/elongation of the fiber, causing a further increase in energy absorption during pull-out. It is assumed that the change of the fiber geometry from circular to trilobal and the associated increased fiber surface area and higher complexity of the cross-section shape lead to the enhanced anchorage in the cementitious matrix and so enables deformation/elongation of the fiber in addition to its surface roughness. Surprisingly, the applied sizing did not lead to higher pull-out resistance in the case of dynamic loading when compared to that of quasi-static loading. Under quasi-static loading, the pull-out resistance was increased by the sizing, which may be due to superior wetting behavior. Based on these findings, further development of polymer microfibers should focus on defined structured surfaces to improve mechanical interlocking in addition to good mechanical characteristics to induce slip-hardening and high-energy absorption under high loading rates.

## Figures and Tables

**Table 1:** Overview of endless polypropylene fibers manufactured by melt-spinning.

Fiber Type	Target Diameter (µm)	Cross-Section Shape	Surface Treatment	Draw Ratio
C10	10	circular	none	1.6
C20	20	circular	none	1.7
C30	30	circular	none	1.9
C20+	20	circular	none	2.25
C20−	20	circular	none	1.0
T20	20	trilobal	none	1.0
C20+ (PP-sized)	20	circular	PP sizing	2.25

**Table 2:** Composition of the concrete matrix [38].

Components	(kg/m^3^)
CEM I 52.5 R-SR3/NA (Holcim, Switzerland)	1460
Silica fume Elkem 971-U (Elkem, Norway)	292
Quartz sand 0.06–0.2 mm (Strobel Quarzsand, Germany)	145
Superplasticizer Glenium ACE 460 (BASF, Germany)	35
Water	315

**Table 3:** Geometric and mechanical properties of the polypropylene (PP) fibers produced, clamping length 10 mm, strain rate 0.5 min^−1^, * equivalent diameter.

Fiber Type	Effective Diameter (µm)	Young’s Modulus (MPa)	Tensile Strength (MPa)	Strain at Break (%)
C10	12.9 ± 0.6	2.3 ± 0.3	269.9 ± 20.0	147.3 ± 23.6
C20	19.4 ± 0.7	3.4 ± 0.1	348.5 ± 11.2	125.8 ± 9.5
C30	29.5 ± 0.7	3.2 ± 0.2	375.5 ± 16.8	119.3 ± 12.1
C20+	19.4 ± 1.0	4.6 ± 0.2	427.1 ± 14.3	76.3 ± 11.7
C20−	21.5 ± 1.2	2.6 ± 0.2	194.8 ± 9.0	228.8 ± 30.3
T20	(20.7 ± 1.6) *	2.6 ± 0.1	183.5 ± 14.7	215.8 ± 20.5
C20+ (PP-sized)	see C20+	see C20+	see C20+	see C20+

**Table 4:** Average roughness R_a_ and maximum roughness R_max_ values of PP fiber surfaces with increasing drawing ratio determined by AFM.

Fiber Type	R_a_ (nm)	R_max_ (nm)
C20−	2.79 ± 1.56	28.20 ± 20.71
C20	2.38 ± 2.04	27.08 ± 18.63
C20+	5.72 ± 1.82	56.92 ± 8.23

**Table 5:** Advancing contact angle of the PP fibers in pure water (Wilhelmy method).

Fiber Type	Contact Angle (°)
C10	90.5 ± 5.6
C20	95.8 ± 5.2
C30	102.3 ± 2.0
C20+	90.0 ± 2.2
C20−	104.5 ± 7.6
T20	93.4 ± 4.0
C20+ (PP-sized)	87.9 ± 5.2

**Table 6:** DSC evaluation of C20−, C20, and C20+ fibers in comparison to the basic PP material.

Fiber Type	Melting Temperature (°C)	Melting Enthalpy (J/g)	Crystallinity (%)
Basic PP material	161	89.4	43.2
C20−	162	91.5	44.2
C20	164	94.9	45.8
C20+	168	87.3	42.2

**Table 7:** Calculated pull-out work for a displacement of 300 µm (W_300_) for quasi-static and dynamic loading and full pull-out for quasi-static loading (W_total_).

	Quasi-Static		Dynamic
Fiber Type	W_300_ (Nm)·10^−6^	W_total_ (Nm)·10^−6^	W_300_ (Nm)·10^−6^
C20−	8.18 ± 1.82	23.42 ± 6.29	10.20 ± 0.95
C20	8.53 ± 1.45	25.88 ± 7.00	18.31 ± 4.08
C20+	10.12 ± 1.53	26.78 ± 6.70	22.34 ± 4.64
T20	6.38 ± 1.47	17.20 ± 4.24	11.43 ± 1.51
C20+ (PP-sized)	12.03 ± 2.52	38.85 ± 16.42	23.46 ± 4.01
C30	13.91 ± 2.66	41.68 ± 12.40	28.24 ± 3.83

## Data Availability

Data sharing is not applicable to this article.
